# Aflibercept clearance through the drainage system in a rat model

**DOI:** 10.1186/s40942-021-00322-8

**Published:** 2021-09-08

**Authors:** Yariv Keshet, Orly Gal-Or, Michal Schaap Fogler, Karin Mimouni, Meydan Ben Ishai, Dov Weinberger, Assaf Dotan

**Affiliations:** 1 Department of Ophthalmology, Rabin Medical Center, Petach Tikva, Israel; 2 Sackler Faculty of Medicine, Tel Aviv University, Tel Aviv, Israel

**Keywords:** Retina, Aflibercept, Intraocular pressure, Choroidal neovascularization, Rats, Brown Norway, Angle, Clearance

## Abstract

**Background:**

As intravitreal anti-VEGF injections became the mainstay of treatment for many retinal diseases, the cause of a secondary sustained elevated intraocular pressure is still unclear. The aim of our study was to study the clearance of Aflibercept from the anterior chamber angle, in a rat model, to test if an aggregation exists.

**Methods:**

Choroidal neovascular lesions (CNV) were induced in the right eye of 12 brown Norway rats, using indirect laser ophthalmoscope. Intravitreal Aflibercept injection (0.12 mg/3 µl) was performed 3 days after CNV induction. Rats were euthanized at predetermine time intervals of 3, 6, 24 and 48 h post injection, with immediate enucleation for histological analysis with H&E and immunofluorescence staining. Aflibercept molecules were stained with red fluorescence thanks to the formation of the immune complex Aflibercept-Rabbit anti human IgG-Anti rabbit antibodies-Cy3.

**Results:**

Immediately after the injection, a strong fluorescence signal was detected, indicating the presence of Aflibercept in the iridocorneal angle. At 3- and 6-h interval a strong signal of Aflibercept was still seen. Six hours post injection, the signal was highly concentrated in Schlemm’s canal. In the 2 eyes harvested 24 h post Aflibercept injection, red fluorescence signal intensity was decreased in one eye, occupying mainly intra scleral venous plexuses, and absent in the other eye. At 48 h there was no fluorescence signal, confirming complete clearance of Aflibercept.

**Conclusions:**

In our rat model, a complete clearance of Aflibercept from the anterior chamber angle, was seen 48 h after the injection. This finding refutes the theory of possible connection between IOP elevation and mechanical obstruction. Evacuation time of Aflibercept through the angle is of the same magnitude as that of Bevacizumab in the same rat model.

## Introduction

The employment of intravitreal injections to inhibit vascular endothelial growth factor (VEGF) has grown tremendously in recent years. Anti-VEGF agents are in wide use for the treatment of a spectrum of ocular disorders, currently constituting the leading form of treatment in neovascular age related macular degeneration, diabetic retinopathy, and macular edema secondary to retinal vein occlusions [1–3].

Aflibercept is a recombinant fusion protein assembled from VEGF-binding segment, based on the extracellular domains of human VEGF receptors 1 and 2, and a fragment crystallizable segment of the human IgG1 immunoglobulin. Due to its specific structure, it is named “VEGF trap”, and inhibits both VEGF-A and VEGF-B isoforms, as well as placental growth factor.

Evidence from several clinical studies in the past decade have demonstrated that receiving multiple intravitreal anti-VEGF injections may cause a sustained intraocular pressure (IOP) elevation in a subset of patients, despite no previous history of ocular hypertension. Sustained IOP elevation has been described after the administration of either Bevacizumab, Ranibizumab or Aflibercept. However, while the rate of increased IOP after Bevacizumab and Ranibizumab injections was found to be similar, the incidence after Aflibercept injections has been described as significantly lower in different studies [4–10].

Several theories have been proposed regarding the correlation between intravitreal anti-VEGF injections and sustained IOP elevation. One proposed explanation is a mechanical insult caused by the drug to the aqueous outflow channels, including the trabecular meshwork (TM) and Schlemm’s canal (SC) [11, 12].

A recent study from our research group [13] demonstrated the presence of Bevacizumab molecules in the aqueous outflow channels after its injection into the vitreous in a rat model. The immunofluorescence staining signal, used to mark Bevacizumab, showed a gradual decrease over time, resulting in an absence of the signal in the anterior chamber angle at 48 h post injection. The findings in this study suggest that following a single injection, Bevacizumab does not accumulate in the TM. The aim of our study, therefore, was to evaluate the clearance rate of Aflibercept through the aqueous outflow channels in a rat model, determining whether it accumulates in any of the angle structures. Using the same rat model enabled us to compare the rate of Aflibercept clearance to that of Bevacizumab, in order to speculate if different clearance rates can clarify the discrepancy in the rate of IOP elevations between the two drugs.

To the best of our knowledge this is the first study performed to test the clearance rate of Aflibercept through the angle in a rat model.

## Methods

Twelve Brown Norway rats weighting 200–300 g each, were used in this study (Envigo RMS, #3BN01). The animals were cared for, in accordance with the Association for Research in Vision and Ophthalmology statement for the use of animals in ophthalmology and vision research with a protocol approved by the institutional animal care and use committee of Rabin Medical Center. During procedures rats were placed under general anesthesia using intramuscular administration of 50 mg/kg body weight Ketamine hydrochloride and 5 mg/kg body weight Xylazine. We used the protocol described in our previous work [13], as follows:

The rat’s eyes were divided into three groups:

- Test group (CNV induction + Aflibercept injection)—the right eye of each rat.
- Positive control group (Aflibercept injection only)—the left eye of 4 rats.
- Negative control group (No injection)—the left eye of 8 rats.

### CNV induction

On day 0, choroidal neovascularization was induced in the eyes of the test group by indirect diode laser photocoagulation (Iris Medical Oculight SLX System©, Iridex, Mountain View, CA, USA), with the treatment beam set at 810 nm and the aiming beam at 650–670 nm, 450 mW power, 100 ms duration, as described previously [14]. Rats were placed under general anesthesia and pupils were dilated using tropicamide (0.5%) eye drops supplemented with topical anesthesia (0.5% proparacaine hydrochloride). One to four laser applications were applied using a 90 Diopter condensing lens at the 3, 6, 9, and 12’ o’clock positions of the posterior pole, at a distance of 1–2 optic disc diameters, surrounding the optic nerve, until the burn produced acute vapor bubble, indicating rupture of the Bruch’s membrane.

### Aflibercept injection

The vitreous VEGF concentration was expected to increase significantly from baseline, after CNV induction [15]. Intravitreal injection of Aflibercept was performed 3 days after laser photocoagulation (test group and positive control group). Under anesthesia, the pupils were dilated using tropicamide 0.5% eye drops. A 30-gauge Hamilton bevel-tip syringe needle was inserted into the vitreous, 1 mm posterior to the limbus, at about a 45-angle and 3 µl of Aflibercept (0.12 mg/3 µl) was injected.

### Immunofluorescence staining and histological analysis

Immediately after Aflibercept injection and at 3, 6, 24 and 48 h, the animals were euthanized, with the eyes enucleated and immediately fixated in 4% PFA and embedded in paraffin. 5 μm sections were prepared and immune-stained with rabbit anti human IgG (for Immunodetection of Aflibercept) and Mouse anti Rat αSMA (for rat smooth muscle detection).

For immunohistochemistry processing, the sections were incubated for 1 h with primary antibodies: IgG (Rabbit Polyclonal, 269 A, Cell Marque, dilution 1:100) and αSMA (Mouse Monoclonal, 202 M-94, Cell Marque, dilution: 1:150) at RT in a humidity chamber, washed, and incubated with secondary antibodies (Donkey & Rabbit Cy3, 711-165-152, Jackson, USA; Donkey & Mouse Cy2, 715-545-151, Jackson, USA). Aflibercept molecules were stained with red fluorescence thanks to the formation of the immune complex Aflibercept-primary antibodies-secondary antibodies-Cy3. First the fragment crystallizable portion of aflibercept was expected to be ligated by the rabbit anti human IgG, which was then recognized by the anti-rabbit antibodies, chemically ligated to Cy3, a red fluorescence stain. DAPI (BLG-422801, Biolegend, USA) was used as a counterstain. For the purpose of anatomical orientation, Hematoxylin and Eosin staining was performed.

### Microscopy

The staining pattern of the tissue sections was observed under Leica TCS SP5 confocal laser-scanning microscope (Leica Microsystems, Germany). Magnification used—×20, 40×, Zoom 1.

All photos were taken in the same exposure conditions. Hence—signal intensity is comparable between groups.

### 3D Image analysis

3D volumetric Image analysis was performed using Imaris software (Bitplane, Oxford Instruments), demonstrating the location of different structures of our interest in the immunostained slides more clearly.

## Results

**Fig. 1:**
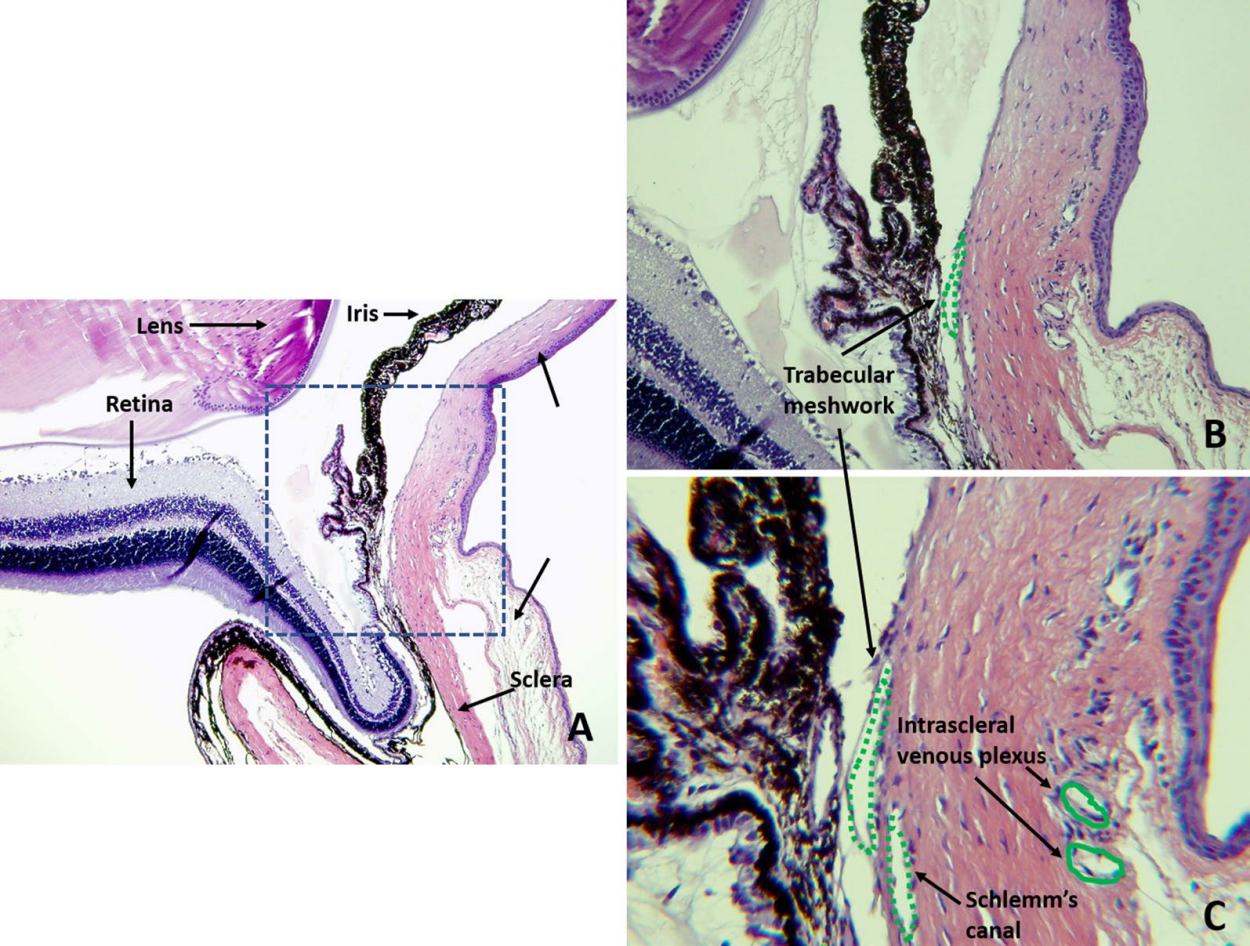
Hematoxylin and eosin staining for anatomical orientation of the anterior chamber angle. **A** ×10, **B** ×20, **C** ×40. structures are clearly visualized as marked in the figure

**Fig. 2:**
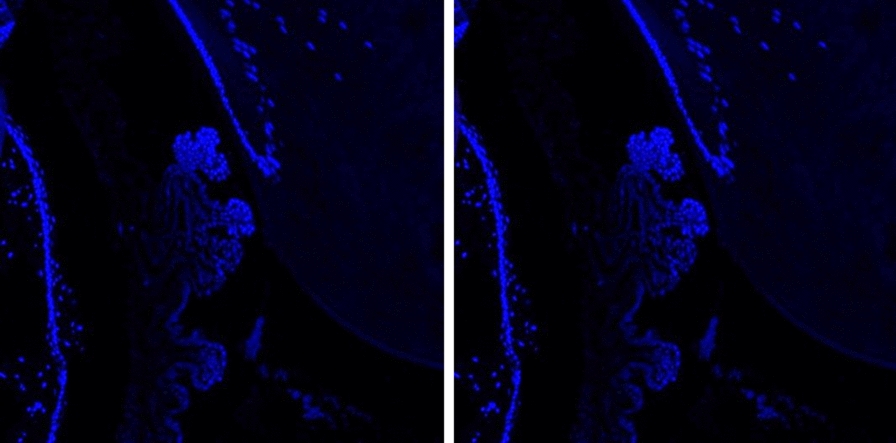
Staining with secondary antibodies only, for the purpose of negative control, demonstrates no staining of Aflibercept molecules or the smooth muscle in any of the slides. **A** Donkey anti Rabbit Cy3. **B** Donkey anti Mouse Cy2

**Fig. 3:**
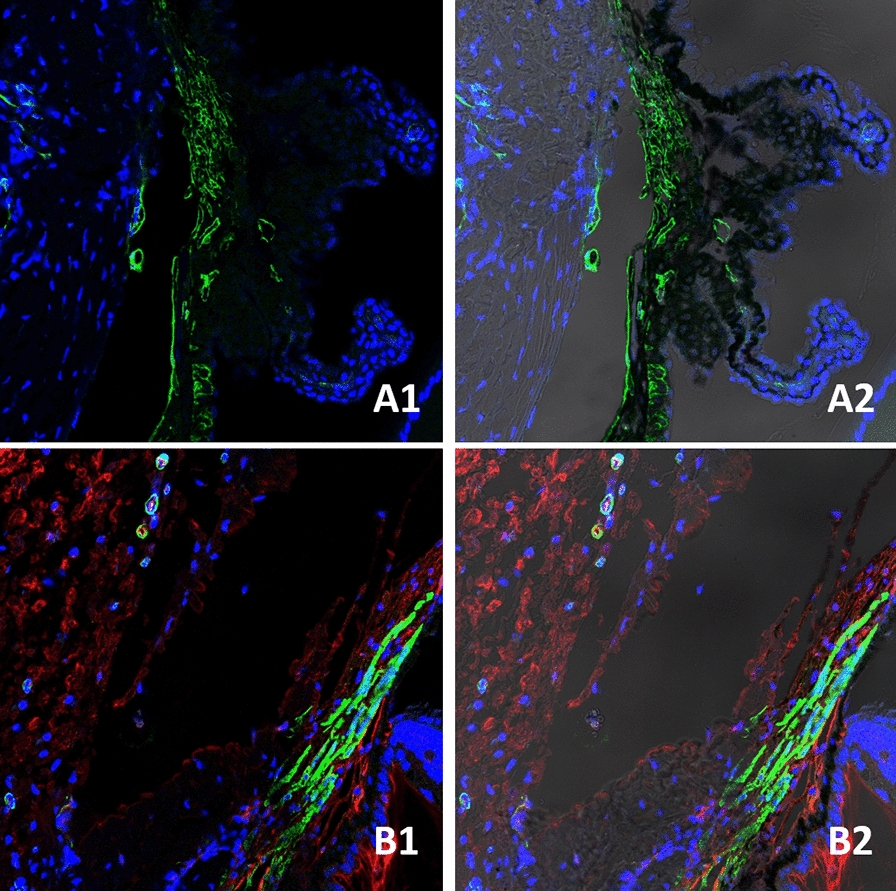
Immunostaining of negative and positive control eyes: Green signal—Mouse Monoclonal anti Rat αSMA, indicating the presence of smooth muscle structures in all controls; Red signal—Rabbit polyclonal anti Human IgG; Blue—DAPI. Right sided photos, 3D reconstruction analysis (Imaris). **A1**–**A2** Negative control, Red signal was not detected. **B1**–**B2** positive control, red signal was detected, indicating the presence of Aflibercept molecules

Twelve Brown Norway rats right eyes were included in the study to learn the clearance of intravitreally injected aflibercept through the iridocorneal angle using immunostaining. The combination of histology and immunostaining profiles were studied and analyzed for the localization of aflibercept molecules. The structures in the anterior chamber angle were clearly visualized and localized with the use of hematoxylin and eosin staining in all study and control eyes, as shown in Fig. 1. Immunostaining using the secondary antibodies solely, Donkey anti rabbit ligated to CY3 (red fluorescence) and Donkey anti mouse ligated to CY2 (green fluorescence), resulted in no staining of Aflibercept molecules or the smooth muscle respectively as shown in Fig. 2. Green fluorescence signal was observed in all eyes, indicating the binding of Mouse anti Rat αSMA to the smooth muscle structures presenting in our slides. The signal was detected in the Iris dilator muscle and Constrictor pupillae, the ciliary muscles, including the longitudinal, radial and circular components and in the walls of blood vessels and angle structures as shown in Fig. 3. Aflibercept molecules bounded by Rabbit anti Human IgG, manifesting as red fluorescence signal, were detected on the iris, cornea and around the angle structures in all positive control eyes. Those molecules were not seen in the negative control eyes, confirming the validity of our immunostaining process.

**Fig. 4:**
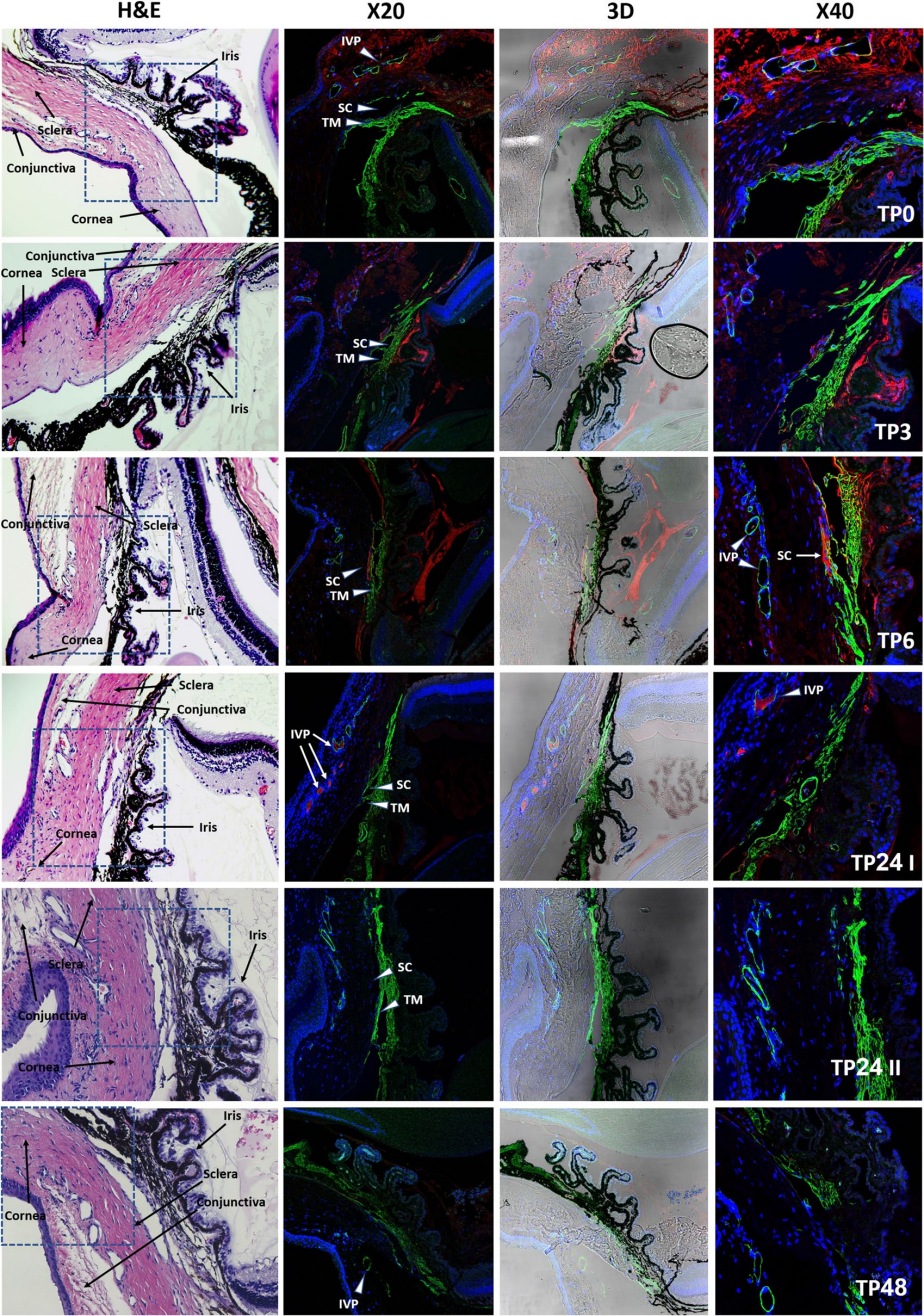
Slides samples from different time points (TP) post intravitreal injections. From left to right are hematoxylin and eosin staining and immunostaining photos with magnification of ×20, 3-dimensional reconstruction and ×40 magnification. At TP 0 Aflibercept molecules are already seen in the anterior chamber angle (red signal). At TP 3, 6 the angle still stains positive for Aflibercept signal, with the signal highly concentrated in Schlemm’s canal at TP 6. In the two eyes at TP 24, only the first one presented still has a red fluorescence signal intensity, albeit decreased, and the other one is missing any signal. At TP 48 no red signal was detected, indicating complete clearance of Aflibercept from the eye. *TM* Trabecular meshwork, *SC* Schlemm’s canal, *IVP* Intrascleral venous plexus

Iridocorneal angle sections prepared at five different time points after aflibercept injection were evaluated. Immediately after the injection, Aflibercept molecules were located around the anterior chamber angle structures, with a strong signal still evident at 3 and 6 h post initial injection time intervals. Six hours post injection, the signal was highly concentrated in Schlemm’s canal. In the two eyes harvested 24 h post Aflibercept injection, red fluorescence signal intensity was decreased in one eye, occupying mainly intra scleral venous plexuses, and absent in the other eye. No red signal was detected in all slides 48 h after the injection, confirming complete clearance of Aflibercept from the eye as shown in Fig. 4.

## Discussion

Intravitreal injections have become one of the most common procedures in the field of ophthalmology and in medicine at large. Sustained IOP elevation after intravitreal anti-VEGF injections has emerged as an important adverse event of these repeated injections. Two recent trials using Ranibizumab or Aflibercept every 4 weeks to treat neovascular age related macular degeneration found a sustained IOP increase of at least 5 mmHg at 96 weeks compared to baseline in 19.7 and 14.1% of eyes, respectively [7]. Various theories regarding the possible mechanism underlying this IOP elevation post intravitreal anti-VEGF injections have been proposed. One theory is a possible mechanical obstruction of the angle structures by protein aggregates [11, 16] or silicone particles [17]. Other possible explanations suggested are drug induced trabeculitis and a possible damage induced to the TM by repeated injections [4, 5, 11, 18, 19]. Nitric oxide pathway and genetic susceptibility may also be involved [9, 20–22].

As more evidence emerges, an obvious distinction is becoming clear between Bevacizumab and Ranibizumab on one hand, and Aflibercept on the other hand. That important distinction may stem from the important differences that exist between Bevacizumab, Ranibizumab and Aflibercept regarding their pharmacodynamics, mechanisms of action and possible targets. Although all three drugs inhibit the ligands (VEGFR-1 and VEGFR-2) from binding to their endothelial receptors, by acting as antagonists, and binding to that exact receptor, Aflibercept has the highest binding affinity of the currently used anti-VEGF drugs. In contrast to Ranibizumab and Bevacizumab which only target the isoform VEGF-A, Aflibercept targets also include PGF and the isoform VEGF-B [23–25]. A study published by Atchison et al. [26] examined IOP increase in a real-world setting. They analyzed 23,776 patients from the IRIS registry, who received injections of Bevacizumab, Ranibizumab or Aflibercept in their right eye, with their fellow eye serving as control. Overall incidents of clinically significant IOP elevation, defined as a rise of at least 6 mmHg that resulted in IOP > 21 mmHg among patients with a baseline IOP ≤ 21, were seen in 2.6% of injected eyes as compared to 1.5% in control eyes. However, while a statistically significant elevated IOP was noted post Bevacizumab and Ranibizumab as compared to the fellow eye, no statistically significant difference was detected in the eyes receiving aflibercept injections. It is worth to mention in this regard, that the group of patients treated with Aflibercept had a higher mean number of injections compared with those treated with Bevacizumab or Ranibizumab (9.3 injections of Aflibercept, 6.9 injections of Bevacizumab and 9 injections of Ranibizumab).

As drugs are injected into the vitreous, their distribution is mechanically decelerated by the aligned collagen and glycosaminoglycans found in the vitreous. Intraocular degradation of any anti-VEGF substances was not shown, so far. Following an intravitreal injection, two possible pathways for the drug to evacuate the eye exists. The first pathway is by crossing the retina and the retinal pigmented epithelium (RPE) to enter the choroidal circulation. The second one is by a passive diffusion of the drug to the anterior chamber, with the elimination process completed by a passage through the TM [27]. As molecular weight of the three above mentioned anti-VEGF injections differs greatly, one may expect their intraocular half-life to behave accordingly. However, this presumption may not necessarily be true. Ranibizumab has the lowest molecular weight of 48 kD followed by Aflibercept weighing 115 kD and Bevacizumab with a molecular weight of 149 kD [28]. Due to the invasive nature of real aqueous half-life measurements of these drugs, only a few studies were performed in vivo. Two studies by Krohne et al. utilized cataract extraction surgery in order to measure the aqueous concentration of Bevacizumab [29] and Ranibizumab [30] at different time intervals after their injection into the vitreous. Their calculated half-life was 9.82 days and 7.19 days for Bevacizumab and Ranibizumab respectively. Presuming the intravitreal half-life of any anti-VEGF injection directly correlates with the molecular weight, Aflibercept would be expected to have a half-life between Ranibizumab and Bevacizumab. However, a recent small case series found the half-life is actually no less than 11 days [31], significantly longer than that of Ranibizumab and of Bevacizumab. There was a certain variability of measured half-life among the patients, which was explained by the authors to possibly derive from differential vitreal liquefaction in different eyes or the presence of additional exit paths in some eyes. The group also speculated that variability in duration of treatment among patients may be related to persistence of high levels of aflibercept in some eyes and not others.

A previous work published recently by members of our current group (G-O.O and D.A) [13] aimed to test the mechanical obstruction theory after Bevacizumab injection. Bevacizumab was injected into the vitreous of similar CNV induced rat model as we used in the present study. Clearance of Bevacizumab molecules through the angle was demonstrated by means of immunofluorescence. Bevacizumab molecules were immunostained using Donkey anti-human IgG, labeled with Alexa Fluor 488, a green fluorescence. Immunoreactivity (IR) for Bevacizumab was shown immediately and 3 h after the injection, primarily in the TM. Six hours after the injection, the TM still showed IR, though less strongly. By 24 h, the fluorescence intensity decreased and mainly located around the episcleral veins. After 48 h, no signal was detected, indicating the complete clearance of Bevacizumab from all anterior chamber angle structures. In the current study, we used the same model in order to further test the obstruction theory following Aflibercept injection. Our results showed similar time interval for clearance of Aflibercept to that of Bevacizumab, with complete disappearance 48 h post injection. This finding of complete drug clearance through the iridocorneal angle after 48 h rebut the theory of possible connection between IOP elevation and mechanical obstruction.

An interesting finding in our study was the complete clearance of Aflibercept in one eye enucleated at the 24-h interval. Although more eyes are necessary for a statistical analysis, this observation contrasted with our previous Bevacizumab study, where green fluorescence intensity was only decreased but not absent, what may suggest a faster clearance time for Aflibercept in our rat model. The half-life levels of anti-VEGF injections varies greatly between different species, an example for that difference can be found in rabbits and monkeys. As opposed to the extended ocular half-life Aflibercept has in humans (11 days), its half-life in rabbits was found to be of the same magnitude to that of Bevacizumab (4.7 days for Aflibercept versus 4.32 days for Bevacizumab) [27]. Another study, published by Park et al., measured the vitreous half-life of aflibercept in rabbits using one compartment model, and found it to be 3.92 days [32], which was significantly shorter than that of Bevacizumab (7.06 days) under the same experimental setting. In addition, the intravitreal half-life in owl monkeys was demonstrated as shorter for Aflibercept as compared to Bevacizumab (2.44 days versus 3.6 days respectively) [33]. To the best of our knowledge, the half-life of Aflibercept or bevacizumab in rats has never been tested.

This work has a few limitations that warrant consideration. It included 12 rats, with a small number of eyes in each group. While the number of eyes did allow us to localize Aflibercept in the angle structures in the different time points, it did not enable a proper statistical analysis. Another limitation lays in the fact that we did not directly compare clearance of Aflibercept to Bevacizumab, but rather relayed on previous results from our group, using the same model. Lastly, only a single intravitreal injection was performed in our study, as opposed to real life multiple repeated injections. Future studies could evaluate whether repeated injections influence the clearance rate differently, with possibly gradual increase in the load of particles in the angle.

In conclusion, we found a complete clearance of Aflibercept signal in the CNV induced rat model in the 48-h time point slides. Those findings further support the notion that other explanations for the evident sustained IOP elevation after Aflibercept injections exists. Further studies are needed to shed more light on the possible causative factors predisposing patients to sustained IOP elevation after intravitreal anti-VEGF injections.

## Data Availability

The datasets used and/or analysed during the current study are available from the corresponding author on reasonable request.
